# Can Preinjury Adversity Affect Postinjury Responses? A 5-Year Prospective, Multi-Study Analysis

**DOI:** 10.3389/fpsyg.2019.01411

**Published:** 2019-06-21

**Authors:** Ross Wadey, Lynne Evans, Sheldon Hanton, Mustafa Sarkar, Helen Oliver

**Affiliations:** ^1^ Faculty of Sport, Health, and Applied Sciences, St Mary’s University, Twickenham, United Kingdom; ^2^ School of Sport and Health Sciences, Cardiff Metropolitan University, Cardiff, United Kingdom; ^3^ School of Science and Technology, Nottingham Trent University, Nottingham, United Kingdom

**Keywords:** coping, emotions, recovery, rehabilitation, stress, trauma

## Abstract

Informed by and drawing on both the integrated model of response to sport injury (Wiese-Bjornstal et al., 1998) and the biopsychosocial model of challenge and threat states (Blascovich, 2008), this multi-study paper examined whether preinjury adversity affected postinjury responses over a 5-year time period. Study 1 employed a prospective, repeated measures methodological design. Non-injured participants (*N* = 846) from multiple sites and sports completed a measure of adversity (Petrie, 1992); 143 subsequently became injured and completed a measure of coping (Carver et al., 1989) and psychological responses (Evans et al., 2008) at injury onset, rehabilitation, and return to sport. MANOVAs identified significant differences between groups categorized as low, moderate, and high preinjury adversity at each time phase. Specifically, in contrast to low or high preinjury adversity groups, injured athletes with moderate preinjury adversity experienced less negative psychological responses and used more problem- and emotion-focused coping strategies. Study 2 aimed to provide an in-depth understanding of *why* groups differed in their responses over time, and *how* preinjury adversity affected these responses. A purposeful sample of injured athletes from each of the three groups were identified and interviewed (*N* = 18). Using thematic analysis, nine themes were identified that illustrated that injured athletes with moderate preinjury adversity responded more positively to injury over time in comparison to other groups. Those with high preinjury adversities were excessively overwhelmed to the point that they were unable to cope with injury, while those with low preinjury adversities had not developed the coping abilities and resources needed to cope postinjury. Practical implications and future research directions are discussed.

## Introduction

For over 20 years, two models have been at the forefront of research into the psychology of sport injury: Williams and Andersen’s (1998) multicomponent theoretical model of stress and injury and Wiese-Bjornstal et al.’s (1998) integrated model of psychological response to the sport injury and rehabilitation process. Underpinned by Williams and Andersen’s model, several preinjury factors have been found to predict injury including personality traits (e.g., hardiness, optimism), adversity (e.g., major negative life events, daily hassles), and coping resources (for a review, see Ivarsson et al., 2017). Postinjury, and consistent with Wiese-Bjornstal et al.’s model, research has also supported the effect of a number of personal and situational factors on athletes’ emotional (e.g., anger, anxiety, guilt, relief) and behavioral responses (e.g., adherence, behavioral coping), and recovery outcomes (e.g., functional performance, readiness to return to sport). However, to date, researchers have largely overlooked the importance of drawing on both models to gain a more complete and comprehensive understanding of the injury process.

According to Wiese-Bjornstal et al.’s (1998) integrated model, preinjury factors that predispose athletes to injury can continue to exert their effects postinjury by influencing injured athletes’ emotional and behavioral responses, and ultimately their recovery outcomes. Indeed, as early as 1995, Wiese-Bjornstal, Smith, and LaMott stated that it would be remiss to think that factors affecting athletes preinjury would simply disappear postinjury. For example, the strain of dealing with a relationship breakdown preinjury was suggested by Wiese-Bjornstal et al. (1995) to likely further compound the strain of dealing with the injury. To date, however, very few empirical studies have examined preinjury *and* postinjury factors; rather, researchers have focused on either preinjury *or* postinjury factors (for notable exceptions, see Albinson and Petrie, 2003; Wadey et al., 2012, 2013). In one of the few studies, Albinson and Petrie examined the effect of a number of preinjury factors (i.e., preinjury adversity, social support satisfaction, and dispositional optimism) on postinjury responses (i.e., appraisals, mood disturbance). Using a prospective methodological design, preinjury measures were completed preseason and postinjury measures were completed 1, 4, and 7 days after injury occurrence. From the 84 Division I-A university football players who completed the preinjury measures, 13 subsequently became injured. Findings identified a positive and significant correlation (*r* = 0.64) between preinjury adversity (i.e., major negative life events) and greater postinjury mood disturbance 1 day after injury occurrence. This finding supported Wiese-Bjornstal et al.’s integrated model and the importance of accounting for preinjury factors when examining postinjury responses.

It is important to recognize that the study by Albinson and Petrie (2003) was not without its limitations. As observed by the authors’ themselves, the sample size was small for a quantitative study (*N* = 13) and sport-specific, reducing statistical power and the potential scope of findings across sports, and they did not account for postinjury responses beyond rehabilitation. According to Albinson and Petrie, to overcome these limitations, researchers should employ multi-site and multisport data collection strategies and account for postinjury responses beyond injury rehabilitation. Another important limitation, and one that researchers need to address, relates to the potential mechanisms underpinning the relationship between preinjury adversity and postinjury mood disturbance. To elaborate, whilst Albinson and Petrie’s finding about the relationship between preinjury adversity and postinjury mood disturbance was intuitively appealing, the authors did not aim to account for, nor seek to explain, the factors and processes underpinning and informing it. Further, Wiese-Bjornstal et al.’s (1998) model offers no theoretical explanation for this relationship because of its descriptive nature rather than theoretical explanation. One theoretical framework that could be used to this end is the biopsychosocial model (BPSM) of challenge and threat states (Blascovich, 2008).

The BPSM (Blascovich, 2008) hypothesizes that prior to a task, individuals will evaluate the demands of the task (i.e., demand evaluation) and whether they possess the necessary resources to cope effectively (i.e., resource evaluation). When an individual evaluates he or she has sufficient resources to meet the demands of the task, a challenge state occurs. In contrast, when an individual evaluates they do not possess the resources required to meet the demands of the task, a threat state occurs (Seery, 2011). The BPSM proposes that these evaluations trigger distinct cardiovascular responses (Blascovich, 2008). To elaborate, a challenge state results in sympathetic-adrenomedullary activation, which releases catecholamines that dilate the blood vessels, and increase cardiac activity and oxygenated blood flow to the brain and muscles. A threat state also results in pituitary-adrenocortical activation, which releases cortisol that inhibits dilation of the blood vessels and reduces cardiac activity, resulting in less blood flow. Consequently, compared to a threat state, a challenge state is marked by relatively higher cardiac output and lower total peripheral resistance (i.e., net constriction vs. dilation in the arterial system; Seery, 2011).

Over the past decade, research has shown a challenge state to facilitate, whereas a threat state to hinder, performance (Hase et al., 2019). Aligned with this research, the BPSM has been used to investigate the relationship between prior adversity and subsequent responses (Seery et al., 2010a,b, 2013). In one such study, Seery et al. (2013) investigated participants’ histories of adversity before a computer-based navigation task. A curvilinear relationship was identified, with a moderate number of adverse life events related to a cardiovascular response more reflective of a challenge state compared to no or a high number of events. In the only study to investigate this assertion in a sport context, Moore et al. (2018) explored the relationship between nonsporting adverse events and cardiovascular responses to, and performance during, a pressurized sporting task. Participants who reported a moderate number of adverse life events displayed cardiovascular responses more reflective of a challenge state compared to those who reported a lower or higher number of events. In addition, participants with a moderate history of adverse events outperformed those who reported a lower or higher number of events. Thus, this contradicts the perspective that adversity increases the risk of future psychological concerns (Turner and Lloyd, 1995). Rather, the findings suggest that exposure to some negative adversity may have a “silver lining.” Specifically, they may benefit individuals during future challenging situations by helping individuals to view such situations as less demanding and/or by enhancing their ability to cope. However, among the limitations of the studies in this area to date are the cross-sectional nature of research designs, a focus on laboratory-based experiments, and the investigation of a limited number of outcomes (e.g., cardiovascular responses, performance). To the best of our knowledge, no research has longitudinally examined the relationship between adverse life events and subsequent responses during and after the experience of a commonplace sporting challenge such as injury. The purpose of this study is to address this oversight by investigating whether preinjury adversity affects postinjury responses (i.e., psychological responses and coping strategies).

## Study 1

Study 1 aimed to extend previous research by providing a 5-year prospective, repeated measures examination of the relationship between preinjury adversity and postinjury responses (i.e., emotional responses and coping strategies). Informed by and drawing on both the integrated model of response to sport injury (Wiese-Bjornstal et al., 1998), the biopsychosocial model of challenge and threat states (Blascovich, 2008), and associated research (e.g., Moore et al., 2018), the following four hypotheses were proposed: (1) injured athletes with a moderate number of preinjury adverse life events would experience less postinjury negative psychological responses at injury onset, rehabilitation, and return to sport compared to injured athletes with a low or high number of preinjury adverse life events; (2) injured athletes with a moderate number of preinjury adverse life events would experience more intense postinjury positive psychological responses at injury onset, rehabilitation, and return to sport compared to injured athletes with a low or high number of preinjury adverse life events; (3) injured athletes with a moderate number of preinjury adverse life events would use more postinjury problem-focused and emotion-focused coping strategies at injury onset, rehabilitation, and return to sport compared to injured athletes with a low or high number of preinjury adverse life events; and (4) injured athletes with a moderate number of preinjury adverse life events would use less postinjury avoidance coping strategies at injury onset, rehabilitation, and return to sport compared to injured athletes with a low or high number of preinjury adverse life events.

### Method

#### Research Design

Scholars have questioned the methodological rigor of research in the psychology of sport injury (e.g., Petrie and Falkstein, 1998; Brewer, 2010). Responding to calls for future research to utilize rigorous methodological designs that have multiple data collection points to account for the temporal nature of recovery (viz. Evans et al., 2006), this study employed a prospective, repeated measures design that aligned with the purpose of the study.

#### Participants

The participants^1^ (*N* = 846) were drawn from six Universities and represented eight team and 18 individual sports and competitive levels that ranged from recreational to international standards of performance. Mean age was 20 (SD = 2.11 years) and 481 were males and 365 were females. Participants’ injury status was monitored for 5 years and 143 subsequently became injured. All injuries were diagnosed by a medically qualified practitioner and included fractures, dislocations, strains, and sprains of different body parts. The resulting time loss from training and competition ranged from 14 to 393 days (*M* days = 41; SD = 50). The injured participants represented team and individual sports from recreational to international standards of competition. Mean age was 19 (SD = 1.21 years) and 76 were males and 67 were females.

#### Measures

##### Preinjury Adversity

The Life Events Survey for Collegiate Athletes (LESCA) was used to measure negative major life events (Petrie, 1992). The LESCA comprises 69 major life events (e.g., death of a close family member, breaking up with partner, failing an important exam, not attaining personal goals in sport, major mistakes in actual competition, being dropped from the team). Participants rated the perceived impact and desirability of each event they had encountered in the last 24 months on an 8-point Likert scale, anchored at −4 (*extremely negative*) and +4 (*extremely positive*). Petrie (1992) reported 1-week test-retest reliabilities ranging from 0.76 to 0.84, and 8-week test-retest reliabilities ranging from 0.48 to 0.72 for the LESCA. Petrie also provided strong evidence of predictive, discriminant, and convergent validity. Only the negative major life events score was used in this study. Participants were divided into low, moderate, and high preinjury adversity groups based on percentile scores. The rationale for this approach was threefold: (1) it aligned with the study’s theoretical underpinning (i.e., biopsychosocial model of challenge and threat states; Blascovich, 2008), (2) it was congruent with our hypotheses, and (3) it has been adopted in previous empirical research (e.g., Moore et al., 2018).

##### Postinjury Coping Strategies

A situation-specific version of the Coping Orientation to Problems Experienced (COPE; Carver et al., 1989) scale was used postinjury to assess coping at injury onset, rehabilitation, and return to sport. The COPE comprises 52 items and 13 different coping strategies (four items per strategy). Participants responded to each item on a 4-point Likert scale, from 1 (*I am not doing this at all*) to 4 (*I am doing this a lot*). Cronbach’s alpha coefficients ranged from 0.52 to 0.90 in this study. Consistent with conceptual models of coping (Hoar et al., 2006) and empirical findings (Stowell et al., 2001; Litman, 2006), the 13 coping strategies were summated into three higher order factors: (1) *problem-focused coping* (i.e., positive reinterpretation and growth, planning, active coping, suppression of competing activities, restraint coping, and acceptance); (2) *emotion-focused coping* (i.e., seeking social support for emotional reasons, focus on and venting of emotions, and seeking social support for instrumental reasons); and (3) *avoidance coping* (i.e., behavioral disengagement, denial, and mental disengagement). The strategy *turning to religion* was excluded from the study on the basis that researchers have demonstrated its failure to load onto any factors (Stowell et al., 2001; Litman, 2006).

##### Postinjury Psychological Responses

The Psychological Responses to Sport Injury Inventory (PRSII) was used to measure athletes’ postinjury psychological responses (Evans et al., 2008). It consists of six subscales: devastation, dispirited, reorganization, feeling cheated, restlessness, and isolation. Each subscale has four items, apart from Reorganization which consists of three items. Participants indicated the extent to which each statement reflected how they presently feel on a 5-point Likert scale anchored at 1 (*strongly disagree*) and 5 (*strongly agree*). Each subscale score ranges from a low of 4 to a high of 20. For Reorganization, this equates to a low of 3 and a high of 15. Evans et al. (2008) provided evidence of content and predictive validity.

#### Procedure

Ethical approval for this study was sought and granted by the first author’s University Research Ethics Committee. Asymptomatic participants (i.e., non-injured and engaging in full participation in sport) were then recruited by approaching key stakeholders (e.g., coaches, lecturers) at recognized sports institutions within the United Kingdom that had large cohorts of competitive athletes. The key stakeholders granted permission that the first author could contact their athletes to request their participation in the study. Group sessions were then undertaken at each institution to explain the aim and scope of the study. Given the longitudinal nature of the study (i.e., 5-year time span), some athletes declined to participate because they were either moving to a new country or ceasing their participation in sport. The athletes who agreed to participate provided written informed consent. Participation was entirely voluntary, and the performers were not compensated in anyway. Participants subsequently completed a demographic information sheet and a preinjury major life event measure (i.e., LESCA), which took 15 min to complete. These were completed online to avoid missing data, according to the standardized instructions recommended by Petrie (1992).

The authors monitored and recorded the injury status of the original sample for a period of 5 years by contacting them and key stakeholders (e.g., coaches, physiotherapists) on a weekly basis after scheduled training sessions or competitions. Consistent with Wadey et al. (2012), an injury was defined as a medical problem resulting from sport participation that prevented normal training and competition for a minimum period of 2 weeks. Minor scrapes and bruises that may require certain modifications (e.g., strapping or protective garments) for training and competition purposes were not classified as injuries. The rationale for not including injuries less than 2 weeks was because this study was interested in postinjury responses at different times phases of recovery (i.e., injury onset, rehabilitation, and return to sport), which injuries of a minimal of 2 weeks’ time loss allowed for (cf. Wadey et al., 2012). Our decision not to focus only on more severe injuries (e.g., a minimum of 6 weeks and beyond; Bianco et al., 1999) was predicated on the need to maximize sample size to increase statistical power. Sample size is a perennial problem with injury research (Cupal, 1998).

If an athlete became injured, they completed the PRSII and COPE at three time points: (1) in the first week of their injury occurrence (i.e., Time 1), (2) midway through their rehabilitation (i.e., Time 2), and (3) in the first week of their return to full training (i.e., Time 3). Questionnaires took 20 min to complete. During the first time point, four other details were also recorded: (1) date of injury occurrence; (2) type and location of the injury; (3) who diagnosed the injury; and (4) estimated duration for recovery (i.e., the approximated number of weeks the athlete would be injured and unable to participate in normal training and competition). The latter information was used to estimate the subsequent two time points for postinjury measure completion (i.e., rehabilitation and return to competitive sport), which was monitored and confirmed by the first author as the participants’ rehabilitation progress unfolded. Postinjury measures included standardized instructions from Evans et al. (2008) and Carver et al. (1989) and were counterbalanced (i.e., ordered randomly).

#### Data Analysis

Data analysis involved three stages. First, data screening procedures were conducted. Second, preliminary analyses were conducted to examine possible differences between groups (i.e., low, moderate, and high preinjury adversity) for demographic factors (i.e., age, sex, and injury severity). These preliminary analyses were used to identify potential covariates. Third, one-way multivariate analysis of variance (MANOVA) was used to explore differences between groups (i.e., low, moderate, and high preinjury adversity) for dependent variables (i.e., coping strategies and psychological responses) at Time 1 (injury onset), Time 2 (rehabilitation), or Time 3 (return to sport). Due to the independent variable having three levels, follow-up one-way analysis of variance (ANOVA) was conducted to identify where significant differences lay. Data analysis was performed using SPSS 21.0 for Windows.

### Results

#### Preliminary Analyses

Three preliminary analyses were conducted to examine differences between groups (i.e., low, moderate, and high adversity) for age, injury severity, and sex. A one-way between-groups analysis of variance (ANOVA) identified no significant difference between groups for age [*F*(2, 140) = 2.3, *p* = 0.103] or injury severity [*F*(2, 140) = 0.1, *p* = 0.990]. Using a Pearson’s chi-squared test, it was identified that there was no statistically significant association between sex and adversity groups, *χ*(1) = 2.270, *p* = 0.321. Demographics therefore were not controlled for in the main analysis.

#### Injury Onset

A one-way MANOVA identified a statistically significant difference at injury onset between groups, *F*(18, 264) = 4.32, *p* < 0.001; Wilks’ Lambda = 0.61, *η*^2^ = 0.23. Using a Bonferroni-adjusted alpha level of 0.005 for the multiple analyses, one-way ANOVAs indicated a significant difference for the following five dependent variables: dispirited, *F*(2, 140) = 13.8, *p* < 0.001, *η*^2^ = 0.23. *Post hoc* comparisons (Tukey’s HSD test) indicated that the high preinjury adversity group (*M* = 13.38, SD = 3.01) was significantly more dispirited than the low (*M* = 11.44, SD = 2.29) and moderate groups (*M* = 10.45, SD = 2.83); Devastation, *F*(2, 140) = 15.6, *p* < 0.001, *η*^2^ = 0.18. *Post hoc* comparisons indicated that the high (*M* = 13.16, SD = 2.55) and low preinjury adversity group (*M* = 12.09, SD = 1.87) reported significantly more devastation than the moderate group (*M* = 10.55, SD = 2.48); problem-focused coping, *F*(2, 140) = 10.6, *p* < 0.001, *η*^2^ = 0.13. *Post hoc* comparisons indicated that the moderate preinjury adversity group (*M* = 54.28, SD = 9.94) reported significantly more problem-focused coping than the high (*M* = 45.11, SD = 12.62) and low groups (*M* = 44.69, SD = 12.82); emotion-focused coping, *F*(2, 140) = 9.3, *p* < 0.001, *η*^2^ = 0.18. *Post hoc* comparisons indicated that the moderate preinjury adversity group (*M* = 26.74, SD = 8.81) reported significantly more emotion-focused coping than the high (*M* = 21.33, SD = 6.99) and low groups (*M* = 20.89, SD = 6.32); and avoidance coping, *F*(2, 140) = 17.4, *p* < 0.001, *η*^2^ = 0.20. *Post hoc* comparisons indicated that the moderate preinjury adversity group (*M* = 18.47, SD = 4.34) reported significantly less avoidance coping than the high (*M* = 27.29, SD = 9.91) and low groups (*M* = 23.98, SD = 7.68). There were no significant differences between groups at injury onset for restlessness, reorganization, isolation, or feeling cheated.

#### Rehabilitation

A one-way MANOVA identified a statistically significant difference at rehabilitation between groups, *F*(18, 264) = 5.38, *p* < 0.001; Wilks’ Lambda = 0.54, *η*^2^ = 0.27. Using a Bonferroni-adjusted alpha level of 0.005 for the multiple analyses, one-way ANOVAs indicated a significant difference for the following dependent variables: dispirited, *F*(2, 140) = 14.5, *p* < 0.001, *η*^2^ = 0.17. *Post hoc* comparisons indicated that the high preinjury adversity group (*M* = 10.87, SD = 2.61) was significantly more dispirited than the low (*M* = 8.27, SD = 2.79) and moderate groups (*M* = 8.26, SD = 2.66); devastation, *F*(2, 140) = 12.87, *p* < 0.001, *η*^2^ = 0.15. *Post hoc* comparisons indicated that the high (*M* = 9.44, SD = 2.93) and low preinjury adversity groups (*M* = 9.00, SD = 2.36) reported significantly more devastation than the moderate group (*M* = 7.21, SD = 1.69); reorganization, *F*(2, 140) = 17.6, *p* < 0.01, *η*^2^ = 0.20. *Post hoc* comparisons indicated that the moderate group (*M* = 10.39, SD = 2.26) reported significantly more reorganization than the high (*M* = 7.98, SD = 1.97) and low groups (*M* = 8.78, SD = 1.91); restlessness, *F*(2, 140) = 14.0, *p* < 0.001, *η*^2^ = 0.17. *Post hoc* comparisons indicated that the high preinjury adversity group (*M* = 10.42, SD = 3.01) reported significantly more restlessness than the low (*M* = 7.80, SD = 2.36) and moderate groups (*M* = 8.08, SD = 2.43); problem-focused coping, *F*(2, 140) = 12.7, *p* < 0.001, *η*^2^ = 0.15. *Post hoc* comparisons indicated that the moderate preinjury adversity group (*M* = 58.32, SD = 12.0) reported significantly more problem-focused coping than the high (*M* = 46.27, SD = 14.24) and low groups (*M* = 46.91, SD = 14.26); emotion-focused coping, *F*(2, 140) = 8.9, *p* < 0.001, *η*^2^ = 0.11. *Post hoc* comparisons indicated that the moderate preinjury adversity group (*M* = 27.66, SD = 9.55) reported significantly more emotion-focused coping than the high (*M* = 23.11, SD = 6.25) and low groups (*M* = 21.35, SD = 6.39); and avoidance coping, *F*(2, 140) = 17.4, *p* < 0.001, *η*^2^ = 0.20. *Post hoc* comparisons indicated that the moderate preinjury adversity group (*M* = 18.47, SD = 4.34) reported significantly less avoidance coping than the high (*M* = 27.29, SD = 9.91) and low groups (*M* = 23.98, SD = 7.68). There were no significant differences between groups at rehabilitation for isolation or feeling cheated.

#### Return to Sport

A one-way MANOVA identified a statistically significant difference at return to sport between groups, *F*(18, 264) = 4.53, *p* < 0.001; Wilks’ Lambda = 0.58, *η*^2^ = 0.24. Using a Bonferroni-adjusted alpha level of 0.005 for the multiple analyses, one-way ANOVAs indicated a significant difference for the following dependent variables: reorganization, *F*(2, 140) = 10.7, *p* < 0.001, *η*^2^ = 0.13. *Post hoc* comparisons indicated that the moderate group (*M* = 11.57, SD = 1.67) reported significantly more reorganization than the high (*M* = 9.64, SD = 2.39) and low groups (*M* = 10.13, SD = 2.40); restlessness, *F*(2, 140) = 10.8, *p* < 0.001, *η*^2^ = 0.13. *Post hoc* comparisons indicated that the high (*M* = 10.58, SD = 3.80) and low (*M* = 9.93, SD = 3.67) preinjury adversity groups reported significantly more restlessness than the moderate group (*M* = 7.79, SD = 1.67); problem-focused coping, *F*(2, 140) = 11.33, *p* < 0.001, *η*^2^ = 0.14. *Post hoc* comparisons indicated that the high (*M* = 58.13, SD = 10.6) and moderate preinjury adversity groups (*M* = 57.19, SD = 13.32) reported significantly more problem-focused coping than the low group (*M* = 46.56, SD = 14.51); avoidance coping, *F*(2, 140) = 10.8, *p* < 0.001, *η*^2^ = 0.12. *Post hoc* comparisons indicated that the high (*M* = 18.93, SD = 3.19) and moderate preinjury adversity groups (*M* = 19.62, SD = 3.19) reported significantly less avoidance coping than the low group (*M* = 23.77, SD = 8.36). There were no significant differences between groups at return to sport for dispirited, devastation, feeling cheated, isolation, and emotion-focused coping.

### Discussion

Aligned with the study’s hypotheses, this study found that athletes with moderate preinjury adversity responded more adaptively postinjury over time than those with lower or higher preinjury negative adverse events. By adaptively, we mean athletes with moderate preinjury adversity not only responded with lower negatively toned psychological responses (i.e., feelings of devastation, dispiritedness, and restlessness) and used less maladaptive coping strategies (e.g., denial, mental disengagement), but they also experienced more positively toned psychological responses (i.e., reorganization) and used greater problem- and emotion-focused coping strategies (e.g., planning, active coping, focus on and venting of emotions) than those with lower or higher preinjury adversity. Findings support Endler and Hunt (1966) and Endler and Magnusson (1976) work on person-situation interactions and Wiese-Bjornstal et al.’s (1998) integrated model, which proposes that preinjury factors affect postinjury responses. However, this model does not stipulate the nature of the relationship between prior adverse life events and subsequent responses to sport injury. Extending the integrated model and associated research (Albinson and Petrie, 2003), the present findings support the notion that exposure to a moderate number of adverse events may have a “silver lining” and may benefit athletes during a future adverse situation such as a sport-related injury – helping them to experience less maladaptive and more adaptive responses in light of prior adversities (cf. Moore et al., 2018).

This study also significantly extends the broader literature on adversity in other fields of research. Indeed, research on the consequences of adversity has long been defined by its traditional focus on the negative effects to health and well-being (e.g., Turner and Lloyd, 1995). The predominant and fundamental assumption of such research is that there is a negative linear dose-response relationship between the extent of adversity experienced and health and well-being. However, the current findings challenge this assumption and provide evidence that adverse experiences may not always be detrimental. Rather, past adverse experiences (e.g., preinjury adversity) can aid future coping with adversity (i.e., sport-related injury). That said, what the current study was not able to explain was *how* moderate preinjury adversity is associated with more adaptive functioning when compared with those lower or higher preinjury adversity. According to the BPSM (Blascovich, 2008), our findings could be explained by those with a moderate preinjury adversity evaluating injury as a challenge because of their prior adversities. In contrast, those with lower or higher prior adversities might evaluate injury as a threat rather than a challenge. To elaborate, Holtge et al. (2018) recently hypothesized higher prior adversities may overwhelm an individual to the point that they are unable to cope with the adversity, whereas a moderate amount of adversity might be sufficiently challenging so that an individual can not only successfully cope, but also learn and improve their coping skills and resources for subsequent exposures to adversity. However, these hypotheses (and others) warrant more research attention to help explain these observed effects.

## Study 2

Building upon the findings from Study 1, Study 2 aimed to provide an in-depth understanding of *why* groups (i.e., low, moderate, and high preinjury adversity) differed in their responses at each time phase (i.e., injury onset, rehabilitation, and return to sport) and *how* preinjury adversity affected these responses. Specifically, it enabled us to explore how preinjury adversity affected athletes’ responses to injury; why do athletes with moderate preinjury adversity respond adaptively postinjury and why do athletes with low or high preinjury adversity respond less adaptively postinjury? Given the richness and complexity required to answer these questions, an ideographic rather than nomothetic methodological design was employed using qualitative inquiry. Considering the research questions were not focused on developing theory (i.e., grounded theory), examining “how” stories are told (i.e., narrative inquiry), exploring conscious experience of everyday life (i.e., phenomenology), or understanding culture (i.e., ethnography), a qualitative tradition was not employed. Rather, this study relied on qualitative methods of data collection to address the participants’ perceptions of *why* and *how* their prior adversities affected their postinjury responses. Several recent reviews illustrate how qualitative methods can achieve these aims (e.g., Culver et al., 2012).

### Method

#### Participants

From the injured athletes (*N* = 143) in Study 1, a two-step procedure was used for Study 2 to select a purposeful sample. Participants’ preinjury major life event scores were used to identify participants who experienced low, moderate, and high preinjury adversity. Those who scored below the 20th percentile were classified as low, those between the 40th and 60th percentile as moderate, and those above the 80% percentile as high. Maximum variation sampling was then used to purposefully sample participants from the three groupings (i.e., low, moderate, and high) to account for predetermined characteristics that would help to offer novel insights into the findings of Study 1, specifically, sex, sport type, competitive level, and severity of injury. This resulted in each group comprising males and females, participants from team and individual sports and different standards of competition, (e.g., recreation, club, regional, national, and international), in addition to injuries that varied in severity. Eighteen injured athletes were contacted, informed about, and invited to participate in the qualitative study; all agreed and provided written informed consent. Of the participants who represented high (*N* = 6), low (*N* = 6), and moderate (*N* = 6) preinjury adversity groups, nine were males and nine were females, who ranged from 21 to 59 years of age (*M*_age_ = 25.4; SD = 9.65). They represented team and individual sports and ranged from club to international levels of performance. At the time of the study, all participants had returned to competition.

#### Interview Guide and Timelining

A semi-structured interview guide developed specifically for this study enhanced the quality of the interviewing process by providing a framework for participants to discuss their experiences while offering the flexibility and freedom for them to share their unique insights, into areas of interest pertinent to the study (Sparkes and Smith, 2014). The interview guide comprised three sections. The first section focused on the participants’ general sporting involvement and the role that injury had played throughout their sporting careers. The aim of this section was to establish rapport with the participants. The second section focused on discussing each of the negative adversities reported in the preinjury questionnaire. Questions included: “Can you tell me more about this event?”; “What (if anything) led up to this event?”; “What impact (if any) did the event have on you?” During this section, the participants were also asked if they had experienced any adversities between completing the preinjury questionnaire and the onset of their injury.

During the second section, to facilitate the interview, timelining was used to visually represent the temporal order of the negative adverse events and how the participants made sense of their experiences over time. The participant drew a temporal graph and plotted the negative events as they unfolded (Sheridan et al., 2011). According to Kolar et al. (2015), timelines can enhance the quality of data collected during interviews by building rapport through actively engaging with the participants.

The third and final section of the interview focused on the effect of preinjury adversities on postinjury responses. This section had three subsections: injury onset, rehabilitation, and return to sport. Example questions included: “Do you think any of the preinjury adversities we’ve discussed impacted your injury experience at this stage of recovery? If so, how? If not, why?” During this stage of the interview, the participants’ quantitative findings from Study 1 were also drawn upon, where appropriate, to facilitate reflection. The interviewer concluded the interview inviting additional insights from the participants.

#### Procedure

Ethical approval for this study was sought and granted by the first and second author’s University Research Ethics Committee. Interviews were conducted face to face by the first and fifth authors in a mutually convenient location. Although the participants were asked the same questions in the same way, each participant’s response determined the sequencing of the questions. This approach was intended to foster a more open communication with participants (Sparkes and Smith, 2014). Elaboration (e.g., “Could you please explain that in more detail?”) and clarification (e.g., “I’m not sure exactly what you meant, could you please go over that again?”) probes were used throughout to elicit more in-depth information and ensure understanding (Sparkes and Smith, 2014). Interviews, which lasted between 80 and 180 min (*M* = 130; SD = 32), were recorded in their entirety and transcribed verbatim. This resulted in over 300 pages of single-spaced transcribed text.

#### Data Analysis and Methodological Rigor

Thematic analysis was conducted by the first author (Braun et al., 2016). The process of analysis initially involved the first author immersing himself in the data by transcribing the data and (re)reading the transcripts multiple times. Initial codes were derived by highlighting interesting features across the entire dataset. Data relevant to each code was subsequently collated and combined to form overarching themes, a process that involved thinking about the relationships between the codes and themes. This involved, for example, exploring horizontal (i.e., themes across the dataset) and vertical (i.e., how themes develop upon one another) patterns within the dataset. To facilitate the process, visual representation (i.e., a thematic map) was used to illustrate the themes and enable the first author to think critically about how the themes related to one another both *horizontally* and *vertically* (Clarke et al., 2017). Themes were then reviewed in relation to the coded extracts, the story they each told, the entire dataset, and the overall story the themes told about the participants’ experiences in relation to the research question.

Throughout this iterative process, a reflexive journal (i.e., introspective reflexivity) was kept by the first author to situate the previous findings from Study 1, his own personal identities, and to explore the surprises and undoings in the research process (i.e., unexpected turns in the research), with himself ultimately becoming the site of analysis and the subject of critique (McGannon and Metz, 2010). These reflections were also shared with the co-authors (i.e., intersubjective reflexivity) at regular intervals. The first author presented his interpretations of the data to them on a regular basis and provided written summaries of the findings for evaluation to enhance the study’s methodological rigor (Smith and McGannon, 2017). The co-authors provided a “sounding board” to encourage reflection upon, and exploration of, alternative interpretations and explanations of the data. As part of this process of critical dialogue, the first author was required to make a defendable case about his interpretations. The production of the final report involved ensuring the write up provided a concise, coherent, logical, non-repetitive, and thought-provoking account of the data, with vivid and compelling example extracts (Braun et al., 2016). In addition, participant reflections on our analytical interpretations were sought (Smith and McGannon, 2017), a process that involved sharing and dialoguing with the participants about the study findings and provided opportunities for additional insight.

### Results

Nine themes were identified in the data that described *how* preinjury adversity affected postinjury responses within each of the three groups and *why* there were differences between them. The results are presented for each group separately (i.e., low, moderate, and high preinjury adversity) and the themes within each group are described in temporal order to align with the vertical thematic analysis. Three themes per group: low (i.e., “Caught in the headlights,” “Not knowing where to turn,” and “Feeling vulnerable”); moderate (i.e., “Looking back to look forward,” “Another challenge to overcome,” and “Coping, recovery, and growth”); and high preinjury adversity (i.e., “The final straw,” “Drained resources,” and “Seeking professional help”). Each theme is now described with illustrative verbatim quotations.

#### Low Preinjury Adversity

##### Caught in the Headlights

This theme was defined as the injured athletes’ shock of being injured and their inability to cognitively process the injury and its short- and long-term implications. Indeed, injury onset was reported to be an overwhelming experience for these athletes, with too many thoughts and emotions to process. One athlete reported, “I just was taken back by it all. I was just in shock that I was injured. There was just so much to get my head around. I didn’t know whether I was coming or going.” Another athlete reported how her reaction was due to having minimal experiences with adversity:

What’s the expression, “A deer caught in the headlights.” I was so shocked that I was injured and, nervous. What have I done? How am I going to get about? When am I going to return? I found it so stressful at the time … The thing is I have not experienced much stress in my life; this was all a new experience for me and I found it tough, really tough. I do not know how to deal with stress.

##### Not Knowing Where to Turn

This theme was defined as the injured athletes’ inability to cope during their rehabilitation from having minimal prior adversities to develop coping abilities and resources. That is, they either lacked coping abilities, used maladaptive coping strategies, and/or did not know how to mobilize their coping resources during rehabilitation. Indeed, the athletes reported continually feeling “lost,” “distressed,” “uncertain,” “confused,” “at a crossroads,” and “not knowing where to turn for help.” One participant reported:

I really struggled during the rehabilitation. I was distraught. I was in pain. I was angry. I was depressed. And I did not know how to deal with these feelings. I’ve never experienced them before… I’ve got lots of friends and family. But, I did not know how to ask others for help. And I did not know who to ask for help. I felt very alone.

One athlete reported using an avoidance coping strategy to manage his negative thoughts and feelings during his rehabilitation. Despite having a short-term desired effect, this strategy proved to be ineffective in the longer term. He reported:

I got really depressed, for a good few months I would say. I was in this, bubble. Every day I’d wake up and I’d have the same brace on my knee and I would say, “Same as yesterday then.” Things were not moving forward. I started gambling, a lot. I missed the buzz of playing rugby (union), so I started to gamble. I gambled every single day for about 3 h. I enjoyed the buzz, it gave me something to do, and it took my thoughts away from the injury … Then, it got out of hand. I became addicted and even more depressed. I needed someone to tell me to step being an idiot.

##### Feeling Vulnerable

This theme was defined as the injured athletes’ reflections upon their return to sport and their ability to cope well. They labeled themselves as “poor copers” and felt vulnerable to future adversity. Looking back, the athletes reported that recovery was a stressful experience, an experience they would not want to reencounter. One athlete reported, “I just can’t cope with stress. Some people can, but clearly, I can’t. I guess you could say I’m a poor coper.” One athlete explained:

I am concerned for the future. I mean, this was my first real experience of stress and I did not handle it well. I guess I’ve lived quite a sheltered life until now. I do not know if I could handle any more stress. This experience has really shaken me. It’s alarmed me. I cannot deal with tough situations. Even sitting here with you now, I feel nervous about any future stresses. I do not know, perhaps I’m not tough enough to make it in sport.

#### Moderate Preinjury Adversity

##### Looking Back to Look Forward

This theme was defined as the injured athletes’ recalling the lessons learnt from their prior adversities and how they could apply them to their current situation. To contextualize this theme, the athletes at injury onset did initially report negative thoughts such as catastrophizing and negative affective states (e.g., depressed, anxious, angry, frustrated). However, they reported over time how they were able to reflect on previous adverse events to regulate these thoughts and feelings, which also reminded them of their personals values and what was important in life. This theme was starkly illustrated by one athlete who reflected on the death of his father to help him rationalize his thoughts and feelings:

I lost my dad. He was in a car with one of my uncles. My uncle was speeding, it was raining, and the car flipped over. A lorry hit the car and my dad died. That’s the biggest thing that has ever happened to me in my life. It made me grow up fast … It’s the moments in life when I need advice, like getting injured, when I really miss him. That’s when it hits you. I got a tattoo 2 years ago, just to remember him. He always used to say, “Together we are strong.” It’s kind of a buffer when I’m feeling down … My dad was always the best person for calming me down. He was as ‘cool as a cucumber.’ But I’ve learnt to do this myself now. I can remember all the things he used to say to me. In that way, he’s never really gone, has he? I’ve become calmer, a more relaxed person. I do not get angry about the little things. Crap happens all the time. You cannot let it bog you down. And I think when I was injured, I started thinking how would dad deal with this? And that played a big part in helping me to come to my senses. It gives me perspective on life really. All I’ve done is hurt my shoulder. I think because other bigger things have happened in my life, I think injury does not seem a big deal any more.

##### Another Challenge to Overcome

This theme was defined as the injured athletes’ appraising injury as a challenge to overcome. Rather than being overwhelmed by the injury experience, these athletes reported injury as an opportunity for growth, development, and mastery. By reflecting upon and recalling the lessons learnt from past and current adverse situations (e.g., loss of parent, parent diagnosed with cancer, miscarriage, friend experiencing a spinal cord injury), the athletes believed they had the coping abilities and resources to deal with the injury. This belief led them to focus on their recovery and how they could keep moving forward rather than dwelling on the past. One athlete reported:

What I’ve learned from past events is that you have got to be positive and not dwell on stuff too much. Focus on the things you can do rather than thinking about the things you cannot do. Yes, I may initially think it’s the end of the world and anticipate the worst but from going through bigger stuff [prior adversities] I soon realise I’m over reacting. I know I can overcome it. You’ve got to put it in perspective and think about how you can make the most out of it. Regardless of whether I’m injured or not, I will fill up my time. That’s my way of coping. How can I make the most of this injury? Could it lead to positive outcomes? It’s just another challenge in life to overcome.

##### Coping, Recovery, and Growth

This theme was defined as the injured athletes’ successful coping efforts to promote their psychological and physical recovery and to ultimately grow from the experience. From experiencing past and current negative adverse events, the athletes reported that they had developed an understanding of how they react to stressful situations and of their coping resources from prior adversities. That is, they knew who to seek support from (and who not to seek support from), to accept support offered from others rather than turning them away, to be proactive rather than reactive, to tell others what support they need rather than letting others determine their support needs, and to not over rely on or tax their resources too much. One athlete reported:

The big thing I’ve learnt from previous events is to talk about how I am feeling. I used to keep my feelings to myself, which made me short tempered and get in to lots of arguments. You can walk around angry all day and hate everyone, but where does that get you? If you just bottle it up it just comes out in other ways. If anything, it’ll make you feel worse and then you will not want to do you physiotherapy. You’re not going to want to get better. I’ve learnt that I need to talk to my family and get everything off my chest. And they also remind me of previous events I’ve faced. I remember talking to my mum about my injury and she reminded me of my friend who became paralysed playing sport. So, my injury wasn’t really the end of the world.

This refined knowledge of themselves and understanding of their coping abilities and resources enabled the athletes to cope with the challenges of rehabilitation and to successfully recover and return to sport. Furthermore, the athletes reported that they learned a great deal from their injury experience. One athlete reported, “Every adverse situation will teach you something. I’ve learnt a lot from the events I’ve experienced in the past, just like I have with this injury.” It was reported that the injury experience reminded them of their values, how mobilizing their social support network had strengthened them, and how they felt more resilient having overcome another stressful experience.

#### High Preinjury Adversity

##### The Final Straw

This theme was defined as how the athletes’ injury was the latest in a series of undesirable events that made them feel that they could not cope with their current situation any longer. The injury was described as the “final straw” and “too much” for them to handle and that they could not keep “spinning the plates” any longer. One participant reported the difficulty with juggling too many adverse life events:

At the same time as the injury, I was dealing with the loss of my father from cancer and we had also just bought a house and we were trying to sell ours. Our buyer pulled out 2 days before completion, and it was just, like everything was going wrong. It, kind of snowballed. It was a really crap time. Not only was my body under a lot of stress, but I was mentally exhausted too. That was a low point and a rough time to go through.

The athletes reported being overwhelmed by what was happening and the only immediate coping strategy reported being used during injury onset was mental disengagement. One athlete reported, “I just denied I was injured and got on the cross-trainer. I needed to vent my feelings, but my injury just got worse.” Another athlete expressed:

Denial, that was my strategy. I would be like, I am not thinking about the injury. It’s a strategy, but it’s bad one because you do not confess to what’s going on and you kind of kid yourself. I put myself in a bubble. And I did not accept anything that was going on … I would just bury myself in other things and try and shut it out.

##### Drained Resources

This theme was defined as venting one’s emotions, ineffective support exchanges, and burdening one’s coping resources. Following the denial of their injury and other adverse situations, the athletes reported during their rehabilitation that their anger and frustrations “boiled” over and they vented onto those in their immediate social network (i.e., family, friends). One athlete reported, “I just couldn’t deny it any longer. It got to the point where I couldn’t suppress my feelings any longer.” Because many of their friends and family were unaware of the athletes’ injury and other past and current adverse events, these revelations came as a shock to them and made for difficult conversations. One athlete reported:

I remember just offloading everything on to my friend. She was taken back by it. She could not keep up with what I was saying. To be honest, I did not really know what I was saying either. I was talking rubbish. I could see she felt uncomfortable and did not know how to respond to me. I just walked off in the end and said not to worry about it, and that I’ll try and figure it out.

These ineffective support exchanges continued as the athletes reported feeling in a “catch-22.” On the one hand, the athletes reported that they did not know what they were thinking and feeling because not only had they not processed the events, but they also had too many events to process. Consequently, they wanted to disclose to others to help process the events and thereby better understand themselves and to let others help them. On the other hand, they found it difficult to articulate all the events and their impact on others, which left members of their support network feeling frustrated from being unable to help. This “catch-22” caused frictions within relationships and led the providers to withdraw their support. One athlete explained: “I had burdened them too much. I could tell they were getting fed up with me. I was getting fed up with me too. I didn’t know where to turn next.”

##### Seeking Professional Help

This theme was defined as seeking help from external sources from taxing their resources and developing symptoms of mental illness (i.e., depression, distress, anxiety). The athletes reported that during the later stages of their rehabilitation and return to competitive sport that they had to seek help from others outside of their social support network. This included support from sport psychologists, psychologists, and/or counselors. One athlete reported, “It got to the point where I needed professional help. I made an appointment with my doctor and he connected me with a psychologist.” At the time of the interviews for this study, many of the athletes reported that they were still receiving professional help. One reported:

I’m still trying to come to terms with all the stuff that’s happened to me, the injury as well. I just could not keep denying it. I needed help. It took me a while to be ready to “open the doors” to how the events have impacted me. Working my way through everything with a psychologist is really helping me to better understand what I’m going through. It’s a horrible process but it’s giving me some perspective.

### Discussion

This study aimed to provide an in-depth understanding of *why* groups (i.e., low, moderate, and high preinjury adversity) differed in their responses over time (i.e., injury onset, rehabilitation, and return to sport) and *how* preinjury adversity affected these responses. Three themes were identified for each of the three groups. For the low preinjury adversity group, the three themes were: “Caught in the headlights,” “Not knowing where to turn,” and “Feeling vulnerable.” These findings provide empirical support for Holtge et al.’s (2018) suggestion that those individuals who experience no or minimal adversities may not develop coping abilities and resources to manage future exposure to adversity. Indeed, the participants reported being overwhelmed when they become injured and that they could not cope. Interestingly, and extending Holtge et al.’s (2018) suggestions in their recent review, this experience also led the participants to report that they felt vulnerable to future adversity. These findings provide somewhat of a dilemma for professional practice. On the one hand, there are increasing recommendations in the literature that to improve the well-being of those involved in sport we should embark on interventions to reduce the likelihood of experiencing adversity (e.g., Randall et al., 2018). These types of interventions are proactive and preventative and based on the assumption that the most effective way to combat the strain experienced by athletes in sport is to eliminate or at least reduce the quantity, frequency, and/or intensity of adverse events. On the other hand, it has been speculated that “talent needs trauma” (Collins and MacNamara, 2012) and the current findings suggest that minimal exposure to adversity does not stimulate the development of coping abilities and resources. Therefore, while not encouraging the experience of negative adverse events, our findings suggest that practitioners should avoid “sheltering” athletes from stressful demands and instead, if suitable, appropriately and progressively optimize the adversities they encounter. In other professions where individuals are required to act under pressure (e.g., police, fire service), exposing individuals to stimulated adversity has facilitated better performance in future stressful scenarios (e.g., Robertson et al., 2015). How best to support athletes who have experienced none or minimal adversity warrants future research.

For the moderate preinjury adversity group, the three themes were: “Looking back to look forward,” “Another challenge to overcome,” and “Coping, recovery, and growth.” To expand, the participants reported they had personally developed from experiential learning with previous adverse situations, which enabled them to view injury as less demanding, believe they can cope given their prior adversities, and evaluate it as a challenge to overcome. From a BPSM perspective (Blascovich, 2008), divergent responses to a pressurized task (e.g., sport injury) are likely due to the differences in how individuals evaluate the task. When resources are judged to match or exceed demands, an individual evaluates a situation as a challenge. When demands are deemed to outweigh resources, an individual evaluates the situation as a threat. This aligns with the present findings, given that the participants had developed their coping abilities and resources from previously experiencing adverse events and as a result they evaluated that they had the resources to cope with their injury. Not only does this finding highlight the importance of injured athletes’ evaluations (cf. Wiese-Bjornstal et al., 1998) and that fostering a challenge state is pivotal to explaining how athletes respond to and recover from injury (cf. Blascovich, 2008), it also reinforces the importance of reflective practice (Ghaye and Ghaye, 1998). Injured athletes should reflect on their prior adverse experiences (and their current injury) as a means of harnessing self-awareness of how they have personally grown from the experience, which aligns with recent research on growth following adversity (Howells et al., 2017) and sport-injury related growth (Roy-Davis et al., 2017).

The concept of growth following adversity in sport is gaining increased research attention. Examples of the types of adversities that have been examined in the sport and performance psychology literature include deselection (Neely et al., 2018); performance slumps, coach conflicts, bullying, eating disorders, and sexual abuse (Tamminen et al., 2013); and repeated non-selection and significant sporting failure (Sarkar et al., 2015). While these adversities have been identified to have negative consequences, the studies have also shown that adversity is not entirely debilitative; it can also bring about positive change, broadly conceptualized as *growth following adversity*. Howells et al.’s (2017) recent systematic review suggested that indicators of growth can be collapsed across three categories: intrapersonal (e.g., new life philosophy, heightened resilience), interpersonal (e.g., less judgmental, increased pro-social behavior), and physical (e.g., superior performance, enhanced body awareness). Yet, while some researchers have examined growth across adversities, others have focused on specific types such as sport-related injuries. Conceptualized as a context-specific form of growth following adversity, Roy-Davis et al. (2017) proposed the term *sport injury-related growth* to reflect the growth that can result from sport injury. Relating back to the current study, the findings support both these conceptualizations. That is, participants reported experiencing growth following various types of preinjury sporting (e.g., major mistakes in actual competition) and nonsporting adversities (e.g., death of a close family member) as well as sport injury specifically. Furthermore, our findings also extend this research by illustrating growth from prior adversities can influence future adverse events. Future research should continue to examine the experience of growth across sporting and nonsporting adversities to further understand the complexity of the phenomenon (cf. Hardy et al., 2017).

For the high preinjury adversity group, the three themes were: “The final straw,” “Drained resources,” and “Seeking professional help.” The participants reported how their injury was the latest in a series of adverse events that made them feel that they could not cope with their current situation any longer. This finding provides empirical support for Holtge et al.’s (2018) suggestion that a high number of adversities may excessively overwhelm the individual to the point that they are unable to cope with the adversity. The participants reported that they did initially try and cope using avoidance strategies (i.e., mental disengagement), which did prove effective in the short term. This supports the findings of Carson and Polman (2010) who identified that avoidance coping strategies postinjury can facilitate control of short-term emotional states. However, our findings suggest that avoidance coping is ineffective in the long term because it can lead to emotional outbursts to others in the athletes’ support network, which can tax the resources of their support providers (cf. Rook, 1992). This finding extends the psychology of sport injury literature. For some time now, social support has been proposed to be a positive way of coping postinjury (for a review, see Bianco and Eklund, 2001). However, it is important to acknowledge that support exchanges can lead to maladaptive responses for the support provider. An important practical recommendation moving forward therefore is that it is not only important to support injured athletes, but it is also critical to monitor and support their social support networks (cf. Wadey et al., 2018). On a final note, the participants in the high preinjury adversity group did report seeking professional help. Clearly, future researchers need to identify interventions that can be used to minimize the damaging consequences of adversity to help athletes cope more effectively with reduced well-being. Examples may include performer assistance programs, clinical counseling, and educational coping programs. Given the rise in mental health concerns in elite athletes (e.g., Souter et al., 2018), this warrants more immediate future research attention.

As with all studies, this study has several limitations that must be acknowledged and accounted for by future researchers to extend this study. First, this study only assessed preinjury adversity once. Given that participants’ appraisal of the desirability of the adversity might have altered over time, future researchers should aim to measure preinjury variables on multiple occasions. Second, there was a time lag between preinjury measures and injury occurrence and this differed between participants. During this time lag, participants may have encountered other adversities and experiences that could have influenced postinjury responses. Third, other preinjury variables were not accounted for that could have explained postinjury findings. For example, the differences in postinjury responses between groups might reflect differences in other personal variables such as mood and the types of experienced adversity rather than the injury *per se*. To account for this in future research, researchers could consider accounting for other preinjury variables, such as mood states as a potential moderating variable. Lastly, the injured samples in this study were heterogeneous in that they differed in the type and severity of injury. This diversity creates challenges for researchers who aim to compare responses across participants at specific time points (e.g., rehabilitation). Future researchers could address this by using more homogenous samples, particularly in relation to injury type (cf. King et al., 2018).

## Conclusion

The studies herein make an important contribution to the psychology of sport injury literature in at least three ways. First, this study is novel in that it is one of the very few studies to integrate preinjury and postinjury factors to help better understand and explain athletes’ responses to injury. Future researchers should continue to examine the interrelationships within the sport injury process (i.e., preinjury to postinjury and back again) to advance this field of research. Second, this study extends our theoretical understanding. Whilst Wiese-Bjornstal et al.’s (1998) integrated model is arguably the dominant model in this field of research, which does hypothesize that preinjury factors may affect postinjury responses, it is descriptive rather than explanatory. The present findings demonstrate that a moderate preinjury adversity can positively influence postinjury responses, whereas higher preinjury adversities can excessively overwhelm the injured athlete and lower preinjury adversities do not challenge the athlete to stimulate the development of coping abilities and resources to enable them to cope with future adversity (e.g., sport injury). It is important, therefore, that future researchers examining adversity not only examine its negative impact, but also understand it can have a “silver lining” and benefit athletes during future adverse situations (Howells et al., 2017). Finally, this study heeded recommendation in the literature (viz. Petrie and Falkstein, 1998; Brewer, 2010) to adopt a rigorous methodology to investigate athletes’ responses to injury (i.e., a prospective, repeated measures, multi-study, multi-method methodological design). In agreement with Brewer (2010), we hope other researchers strive, “… to conduct investigations of the calibre needed to thoroughly examine the role of psychological factors in sport injury rehabilitation outcomes” (p. 57).

## Data Availability

The quantitative dataset is available on request. However, due to the sensitive nature of the interviews, the participants did not grant us permission to share the transcripts from the qualitative interviews. Due to the sensitive nature of the interviews, the participants did not grant us permission to let others read the transcripts. However, they have granted permission for the quotes that have been selected in the article.

## Ethics Statement

This study was carried out in accordance with the recommendations of St Mary’s University’s Research Ethics Committee with written consent from all subjects. All subjects gave written informed consent in accordance with the Declaration of Helsinki. The protocol was approved by St Mary’s University’s Research Ethics Committee.

## Author Contributions

RW contributed to research design, data collection, data analysis, and write up. LE and SH contributed to research design and editing of the manuscript. MS contributed to write up and editing of the manuscript. HO contributed to data collection and editing of the manuscript.

### Conflict of Interest Statement

The authors declare that the research was conducted in the absence of any commercial or financial relationships that could be construed as a potential conflict of interest.

## Footnotes

^1^694 participants were drawn from a previously published study (Wadey et al., 2012); however, the relationship between major life events and postinjury responses was not examined in this study. Furthermore, the current study recruited an additional 170 participants.
